# miR-148a-mediated estrogen-induced cholestasis in intrahepatic cholestasis of pregnancy: Role of PXR/MRP3

**DOI:** 10.1371/journal.pone.0178702

**Published:** 2017-06-02

**Authors:** Zhou-Zhou Rao, Xiao-Wen Zhang, Yi-Ling Ding, Meng-Yuan Yang

**Affiliations:** Department of Obstetrics and Gynecology, The Second Xiangya Hospital, Central South University, Changsha, China; Digestive Disease Research Center, Scott & White Healthcare, UNITED STATES

## Abstract

Intrahepatic cholestasis of pregnancy (ICP) is an idiopathic liver disease while the biochemical characteristic is the elevated level of total bile acid (TBA). The present study investigated whether miR-148a mediates the induced effect of estrogen on the development of ICP and the proper mechanism: PXR/MRP3 signal pathway. mRNA expression was detected by qPCR, protein expression was detected by western blotting, the concentration of estrogen and TBA were detected by reagent kit respectively. In the cinical research, it was found that miR-148a expression was positive related with the concentration of TBA in the serum of ICP patients. In *in vitro* research, estradiol (500 nmol/L, 12 h) significantly upregulated miR-148a expression and LV-148a-siRNA inhibited the function of estradiol (500 nmol/L, 48 h) on TBA secretion. In addition, gene silence of miR-148a upregulated PXR expression which was inhibited by estradiol in LO2 cells. Pretreatment of rifampin (10 μmol/L), the agonist of PXR alleviated the TBA secretion induced by estradiol (500 nmol/L, 48 h). miR-148a-siRNA and PXR had a synergistic action on TBA secretion of LO2. Both of miR-148a-siRNA and rifampin (10 μmol/L) inhibited the upregulated effect of estradiol on MRP3 expression. This research has demonstrated that miR-148a may be involved in the induction of estrogen on ICP via PXR signal pathway, and MRP3 may be involved.

## Introduction

Intrahepatic cholestasis of pregnancy (ICP) is a severe liver disease uniquely occurring during the second and third trimesters of pregnancy [1, 2]. Clinical research confirms that ICP is a risk in perinatal infants as it destroys the structure and function of foetal organs, leading to dysfunction. The previous researches have confirmed that the disease resulted in decreasing rates of premature birth and increaseing rates of postnatal mortality in China population[3]. However, the mechanism of ICP development is still unknown.

The microRNA (miRNA) miR-148a is a 17- to 25-nucleotide (nt)-long highly conserved single-stranded non-coding RNA regulating the expression of target genes at the post-transcriptional level by binding to the complementary sites of the mRNA of specific target genes [4]. Recently, miR-148a was reported to regulate low-density lipoprotein receptor and ATP-binding cassette, subfamily A, member 1 (*ABCA1*) expression to control circulating lipoprotein levels [5]. miR-148a plays a pivotal role in the liver by promoting the hepato-specific phenotype [6]. In addition, miR-148a induces autophagy and apoptosis in hepatic stellate cells through the sonic hedgehog signalling pathway [7]. On the other hand, recent studies have demonstrated that estrogen regulates miRNA expression [8, 9]. Previously, we reported that miR-148a from placenta is associated with the pathogenesis of ICP [10]. Therefore, we speculated that the regulatory effect of miR-148a on hepatocellular TBA secretion may be involved in ICP development.

The pregnane X receptor (PXR, NR1I2) is a ligand-activated transcription factor that belongs to the nuclear hormone receptor (NR) superfamily [11]. PXR is highly expressed in the liver and intestine, but low expression levels have also been found in many other tissues [12]. Importantly, PXR is activated by the toxic bile acid lithocholic acid (LCA), whereas PXR serves as a physiological sensor of LCA, and co-ordinately regulates gene expression to reduce the concentrations of this toxic bile acid [13]. These results suggest that PXR plays an important role in bile acid secretion in the liver. Interestingly, bioinformatics and dual luciferase experiments have identified that miR-148a directly participates in the post-transcriptional regulation and expression of PXR in hepatocytes [14]. Therefore, we considered that miR-148a–induced downregulation of PXR expression contributes to ICP development induced by oestrogen.

Multidrug resistance protein 3 (MRP3) is an ATP-dependent protein located in the basement membrane of hepatocytes that mediates the transport of bile acid from liver cells to hepatic sinuses [15, 16]. In a cell model of MRP3 overexpression, it was demonstrated that MRP3 plays a significant role in the cholehepatic circulation of bile salts [17]. In animal experiments, PXR and MRP3 expression was found to be downregulated in late normal pregnancy with a high estrogen concentration [18, 19].

Based on the above research, we suggested that miR-148a–targeted regulation of the PXR/MRP3 signalling pathway is involved ICP development induced by estrogen.

## Methods and materials

### Clinical subjects

Patients with ICP (n = 25) or normal pregnancy (n = 28) were recruited for analysis. All individuals were patients at Xiangya Second Hospital, Central South University, Changsha. ICP was clearly identified by plasma TBA levels. The healthy subjects, who underwent caesarean section because of an abnormal foetal position, cephalo-pelvic disproportion, umbilical cord factors, or social factors, had no history of gallstones or cholecystopathy, pruritus, drug use, hepatitis, or any other diseases associated with hepatobiliary function. All data were recorded in a computerised database by a research assistant. All participants were Han Chinese from Changsha or the surrounding counties. All subjects signed an informed consent to participate in the study, which was approved by the Ethics Committee of Xiangya Second Hospital.

### Determination of estrogen concentration

Serum from peripheral venous blood was collected to detect the estrogen concentration. Samples from all of subjects were collected after overnight fasting before their caesarean section in the operating theatre. Assessments were performed on a Hitachi 7170A biochemistry analyser (Hitachi, Tokyo, Japan) using a circulating enzymatic method kit (Xinyu Technology Co., Ltd., Shanghai, China).

### Cell culture

LO2 is a normal human liver parenchymal cell line. The cell lines were purchased from the American Type Culture Collection (Rockville, MD, USA) and maintained in Dulbecco's modified Eagle's medium (Gibco, Gaithersburg, MD, USA) supplemented with 10% foetal bovine serum (16000–044, Gibco) and maintained at 37°C in a humidified atmosphere with 5% (v/v) CO_2_.

### Transfection of LV-miR-148a-siRNA

Before transfection of lentiviral (LV)-miR-148a-small interfering RNA (siRNA) (GeneChem, Shanghai, China) for 24 h, LO2 cells were seeded in 24-well plates at 0.4 × 10^5^ cells/well, until their density reached 50%–80%. Cells were then infected with the LV-miR-148a-siRNA using antibiotic-free and serum-free Opti-MEM culture medium. As the reference infection dosage, 2 μL of 1 × 10^9^ transducing units of virus per millilitre were added per well for a multiplicity of infection of 10. Five days after transfection, cells were extracted to perform real-time polymerase chain reaction (RT-PCR).

### RNA isolation and real-time RT-PCR analysis

Total RNA isolation from serum or the cell line was performed using Trizol reagent (Invitrogen, Carlsbad, CA, USA) according to the manufacturer’s instructions. The expression level of mature miR-148a in cells and serum samples was detected by quantitative RT-PCR (qRT-PCR) and calculated as described previously [20]. The expression level of PXR and MRP3 mRNA were measured by qRT-PCR using the SYBR Green Real-time PCR Expression Assay (Takara, Shiga, Japan). The β-actin mRNA level was used for normalisation. The specific primer pairs are shown in Table 1. The relative expression of PXR and MRP3 mRNA compared with β-actin mRNA was calculated using the 2^-ΔCT^ method.

**Table 1:** Sequences of Real-Time PCR Primers.

Primers	Sense
PXR	P+:5’-AGCTGGAACCATGCTGACTT-3’ P-:5’ -CACATACACGGCAGATTTGG-3’
MRP3	P+:5 ‘-AAAAGCAGACGGCACGACA-3’ P-:5 ‘-GCAGGCACTGATGAGGAAGC-3’
β-actin	P+:5’-CTGCACCACCAACTGCTTAG-3’ P-:5’-AGGTCCACCACTGACACGTT-3’

### Western blot analysis

Total proteins were extracted and separated by 10% sodium dodecyl sulphate-polyacrylamide gel electrophoresis and then transferred onto polyvinylidene difluoride membranes (Millipore, Bedford, MA, USA). The blotted membranes were incubated with anti-human PXR antibody (Santa Cruz Biotechnology, Santa Cruz, CA, USA) and anti-human MRP3 antibody (Santa Cruz Biotechnology) at 4°C overnight and then incubated with horseradish peroxidase-conjugated secondary antibody (Santa Cruz Biotechnology) for 1 h at room temperature and detected by chemiluminescence (SuperSignal West Pico Chemiluminescent Substrate; Thermo Scientific, Rockford, IL, USA). β-actin protein determined according to its antibody level (Epitomics Biotechnology) was used as a loading control.

### Statistical analysis

Results are expressed as the mean ± standard error of the mean. Data were analysed using a *t*-test for comparisons of two groups or one-way analysis of variance followed by Tukey’s test for multiple comparisons. Differences were considered statistically significant when *P* < 0.05 where the critical value of *P* was two-sided. Analyses were performed using SPSS version 16.0 (SPSS Inc., Chicago, IL, USA).

## Results

### Relationship of miR-148a and ICP

Table 2 shows the general characteristics of the study participants. Compared with the normal-pregnancy group, patients with ICP had significantly higher estrogen (*P* < 0.001) and TBA (*P* < 0.001) concentrations with a little short of pregnant week (*P <* 0.001). Compared with the normal-pregnancy group, higher serum miR-148a expression was detected. As shown in Table 2, miR-148a was significantly upregulated in the ICP group (*P* < 0.001). The two study groups did not differ with respect to age, amniotic fluid grade, placenta grade, foetal heart monitoring, or APGAR1 or APGAR5 (Table 2). Furthermore, we analysed the correlation of the plasma level in miR-148a, TBA and estrogen. The data shown in Table 3 that there were positive relationship between miR-148a and TBA (p = 0.021), TBA and estrogen (< 0.001), miR-148a and estrogen (p = 0.002). These results suggested that miR-148a expression was positively related to the estrogen and TBA concentrations in patients with ICP.

**Table 2:** Clinical patients characterists.

	Normal group (n = 28)	ICP (n = 25)	P
Age	28.8 ±1.2	28.4 ±0.9	0.378
Pregnant week	38.6 ±0.1	37.4 ±0.2	<0.001
Amniotic fluid index	114.0 ±5.4	117.4 ±4.0	0.620
Amniotic fluid grade	0.54 ±0.20	0.68 ±0.22	0.632
Placenta grade	1.93 ±0.09	2.00 ±0.00	0.469
Fetal heart monitoring	8.39 ±0.19	8.36 ±0.11	0.885
APGAR1	8.9 ±0.1	8.8 ±0.1	0.716
APGAR5	10 ± 0.0	9.9 ±0.1	0.294
Extrogen (pmol/L)	85.7 ± 0.8	112.1 ± 2.	<0.001
TBA(μmol/L)	3.86 ± 0.37	37.49 ± 4.88	<0.001
MiR-148a	0.06 ±0.01	0.30 ±0.07	<0.01

**Table 3:** Relationship of different variables.

Variable	*r*	*P*
Extrogen and miR-148a	0.317	0.021
Extrogen and TBA	0.651	< 0.001
miR-148a and TBA	0.415	0.002

### Effect of estradiol on TBA secretion in LO2 cells

**Fig 1:**
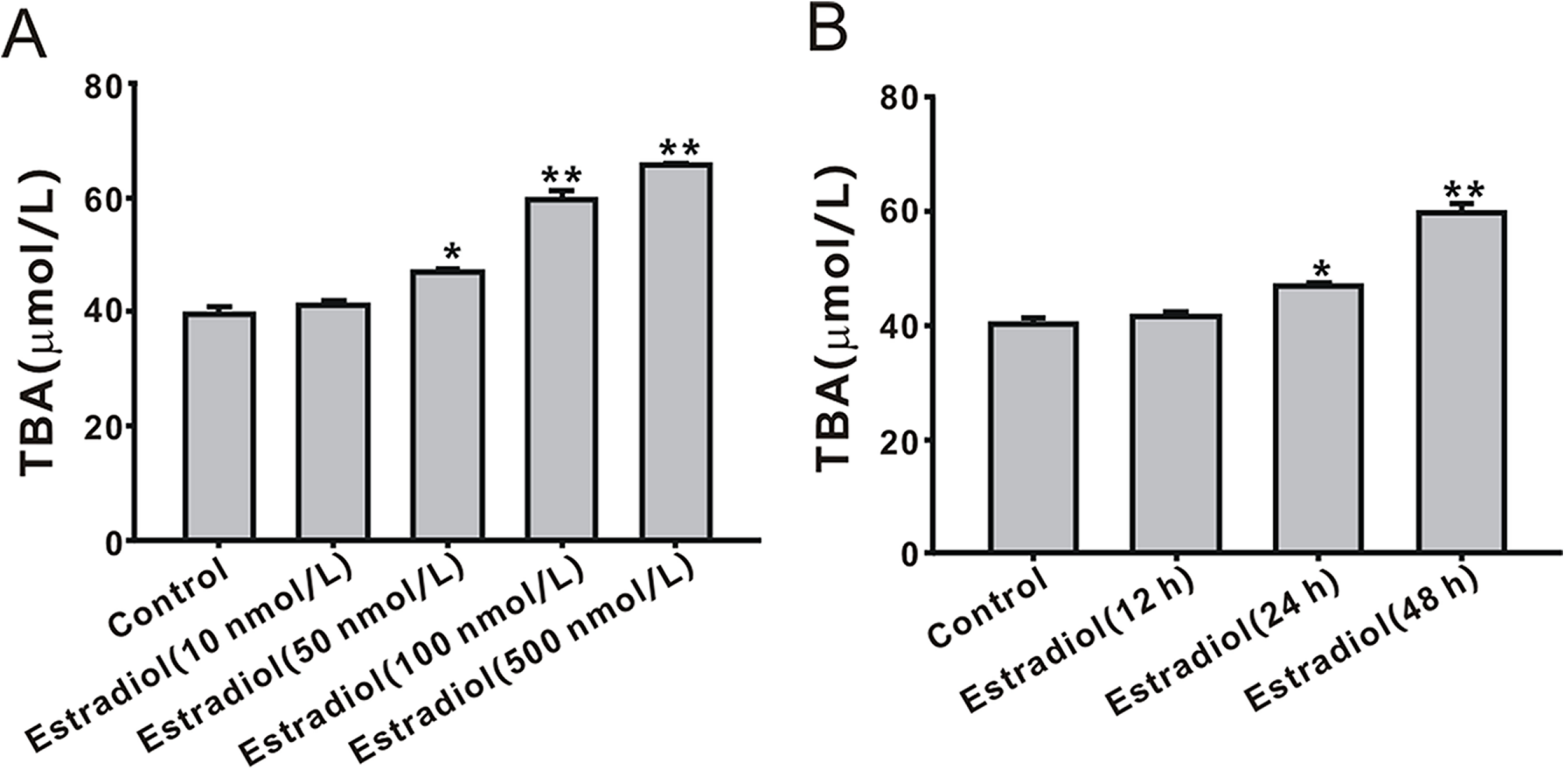
Effect of estradiol on the secretion of total bile acid (TBA) in LO2 cells. (A) TBA secretion was induced by estradiol in a dose-dependent for 24 h. (B) TBA secretion was induced by estradiol in a time-dependent manner. *P < 0.05, **P < 0.01 vs. control group (n = 3).

To explore the relationship of estrogen and TBA, LO2 cells were treated with estradiol at different doses and for different times to mimic the high estrogen concentration *in vitro*. As shown in Fig 1A, estradiol induced an increase in the TBA concentration in a dose-dependent manner, and the effect was significant at 500 nmol/L (1.98 ± 0.07 μM, *P* < 0.01). Treatment of LO2 cells with 500 nmol/L estradiol for different times (12, 24 and 48 h) showed a significant effect on TBA secretion at 48 h (*P* < 0.01, Fig 1B).

### Role of miR-148a in TBA secretion induced by estradiol in LO2 cells

**Fig 2:**
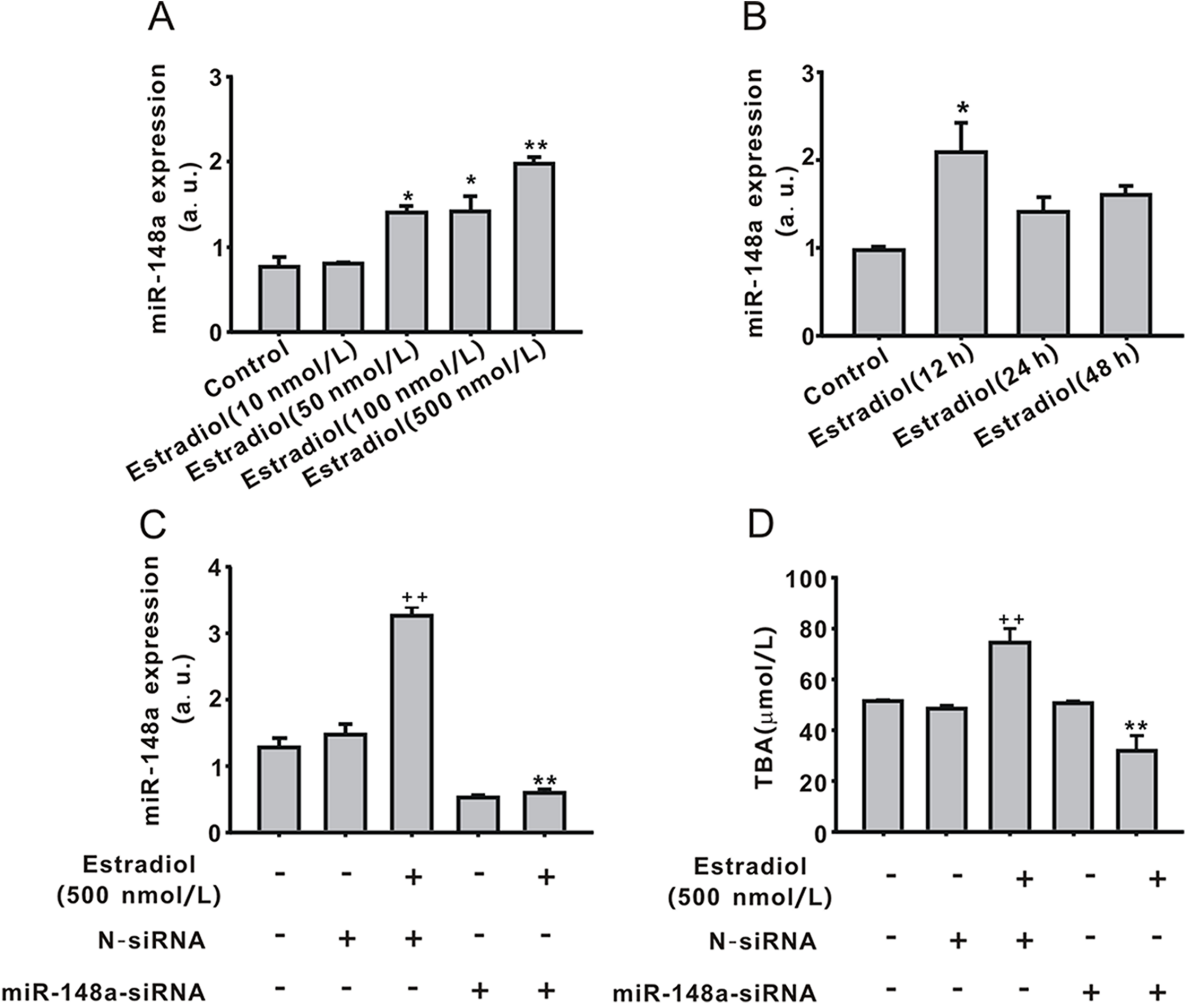
Regulatory effect of miR-148a on total bile acid (TBA) secretion induced by estradiol. (A)The expression of miR-148a was upregulated by estradiol in a dose-dependent for 24 h. (B)The expression of miR-148a was upregulated by estradiol in a time-dependent manner. (C)The transfection of miR-148a-siRNA inhibited the effect of estradiol on miR-148a expression. (D) The transfection of miR-148a-siRNA decreased TBA level in the medium. Values represent the mean ± standard error of the mean (n = 3). **P < 0.01 vs. N-siRNA plus estradiol (500 nmol/L, 12 or 48 h); ^++^*P* < 0.01 vs. N-siRNA.

Compared with control cells, estradiol upregulated miR-148a in a dose-dependent manner, with the 500 nmol/L dose being the most effective (1.98 ± 0.07, *P* < 0.01, Fig 2A). MiR-148a expression was also detected in LO2 cells treated with estrogen (500 nmol/L) for 12, 24, or 48 h. Interestingly, miR-148a was greatly upregulated by estradiol (500 nmol/L, 12 h), but its expression recovered after 12 h (Fig 2B). These results suggest that miR-148a can be regulated by estradiol. Furthermore, to explore the regulatory effect of miR-148a on TBA secretion induced by estradiol, LV-miR-148a-siRNA was used. As shown in Fig 2C, LV-miR-148a-siRNA successfully interrupted miR-148a expression and also inhibited the estradiol-induced upregulation of miR-148a expression compared with N-siRNA plus estradiol (500 nmol/L, 12 h, *P* < 0.01). Estradiol (500 nmol/L, 48 h) increased TBA (74.4 ± 5.9, *P* < 0.01) secretion, and LV-miR148a-siRNA significantly inhibited the estradiol effect on TBA (Fig 2D). LV-N-siRNA exerted no effect on miR-148a or TBA.

### Role of PXR in the regulatory effect of miR-148a on TBA secretion induced by estradiol in LO2 cells

**Fig 3:**
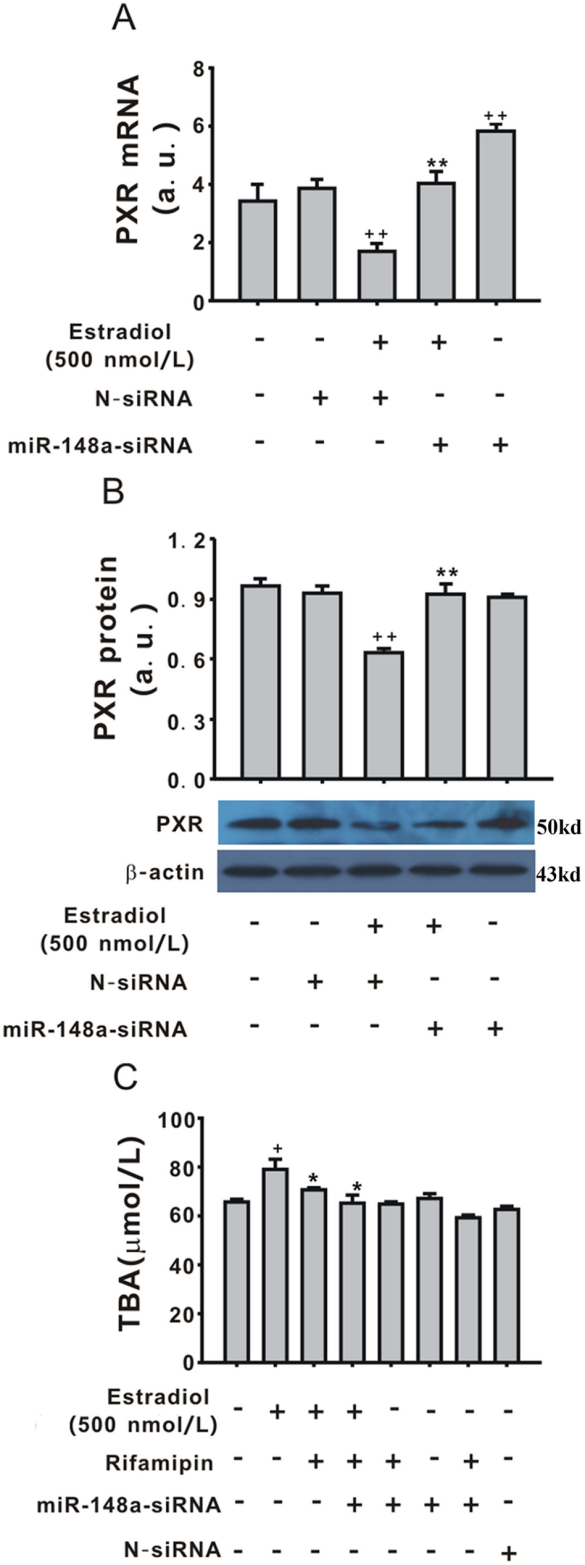
Involvement of pregnane X receptor (PXR) in the effect of miR-148a on total bile acid (TBA) secretion induced by estradiol. PXR mRNA expression was detected by quantitative polymerase chain reaction. (B)PXR the protein expression was detected by western blotting. (C) Rifampin (10 μmol/L) reversed the effect of estradiol on TBA secretion in the medium. Values represent the mean ± standard error of the mean (n = 3). ^+^*P* < 0.05, ^++^*P* < 0.01 vs. control group, *P < 0.05, **P < 0.01 vs. estradiol (500 nmol/L, 12 or 48 h).

To explore the role of PXR in TBA secretion induced by estradiol, PXR expression was detected in LO2 cells treated with estradiol and LV-miR-148a-siRNA. As shown in Fig 3A and 3B, PXR mRNA and protein expression were both significantly downregulated by estradiol (500 nmol/L, 12 h) compared with control (*P* < 0.01), and LV-miR-148a-siRNA reversed this effect (*P* < 0.01). These results implied that PXR was regulated by miR-148a in LO2 cells treated with estradiol. Therefore, to explore the synergistic effect of PXR and miR-148a-siRNA on TBA, the miR-148a–silenced LO2 cells were pre-treated with rifampin (10 μmol/L), a PXR agonist, for 30 min before estradiol (500 nmol/L). As shown in Fig 3C, rifampin (10 μmol/L) inhibited the effect of estradiol (500 nmol/L, 48 h) on TBA secretion. Furthermore, the combination of LV-miR-148a-siRNA and rifampin (10 μmol/L) inhibited TBA secretion induced by estradiol (500 nmol/L, 48 h, Fig 3C). Treatment with rifampin, LV-miR-148a-siRNA, or LV-N or LV-miR-148a-siRNA plus rifampin without estradiol had no effect on TBA secretion (Fig 3C).

### The role of MRP3 in the effect of estradiol in LO2 cells

**Fig 4:**
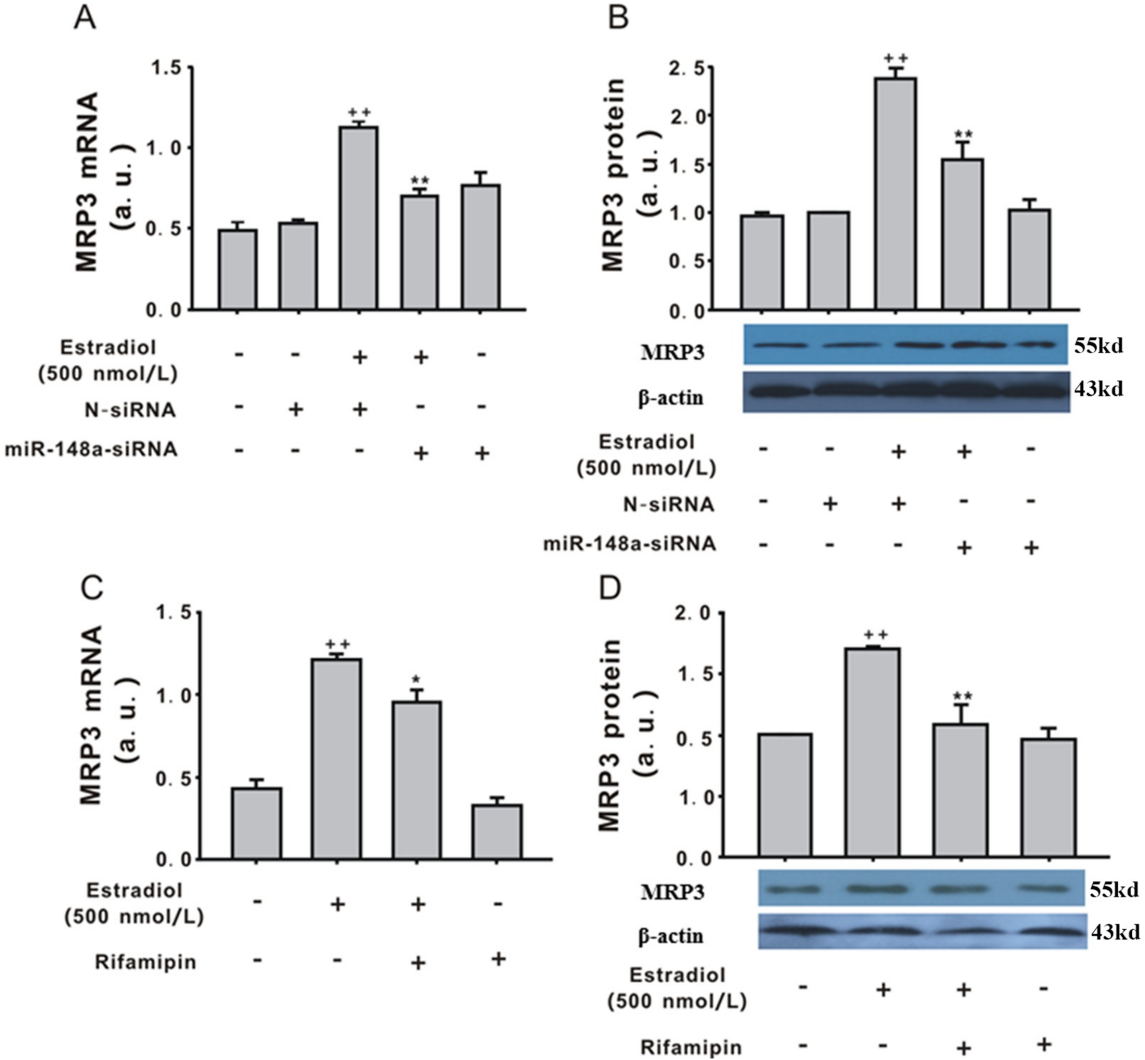
Involvement of MRP3 in total bile acid (TBA) secretion induced by estradiol. (A) LV-miR-148a-siRNA regulated MRP3 mRNA expression induced by estradiol (500 nmol/L, 12 h). (B) LV-miR-148a-siRNA regulated MRP3 protein expression induced by estradiol (500 nmol/L, 12 h). (C) Rifampin (10 μmol/L) reversed the effect of estradiol on MRP3 mRNA expression. (D)Rifampin (10 μmol/L) reversed the effect of estradiol on MRP3 protein expression. Values represent the mean ± standard error of the mean (n = 3). ^+^*P* < 0.05 vs. control group, *P < 0.05, **P < 0.01 vs. estradiol (500 nmol/L, 12 h).

As shown in Fig 4A and 4B, estradiol (500 nmol/L, 12 h) significantly upregulated MRP3 mRNA and protein expression (*P* < 0.01), and miR-148a overexpression inhibited the effect of estradiol on MRP3 expression. LV-N had no effect on MRP3 expression. MiR-148a significantly upregulated MRP3 mRNA expression (Fig 4A) but had no effect on MRP3 protein expression (Fig 4B). To explore the relationship of PXR and MRP3, rifampin was used. Compared with estradiol (500 nmol/L, 12 h), pre-treatment with rifampin (10 μmol/L) partially reversed the effect of estradiol on MRP3 mRNA (Fig 4C) and protein expression (Fig 4D), whereas rifampin (10 μmol/L) alone had no effect on MRP3 expression.

## Discussion

The main findings of the present study are as follows: 1) in the clinical research, serum miR-148a expression was upregulated and positively related to ICP development; 2) estradiol induced TBA secretion in LO2 cells via the upregulated miR-148a expression; and 3) the PXR/MRP3 signalling pathway mediated the effect of miR-148a on TBA secretion.

Deregulated expression of miRNAs has been reported in many human liver diseases [21–23]. Functional characterisation of these miRNAs and their target proteins in tumorigenesis has been important in identifying novel therapeutic targets. miR-148a was first shown to block apoptosis [24], and many studies found that miR-148a expression is closely related to different types of hepatic disease. MiR-148a mediates the development of hepatic cancer via the target gene *PTEN* [25]. MiR-148a also plays a pivotal role in the liver by promoting the hepato-specific phenotype and suppressing the invasiveness of transformed cells by regulating c-Met expression [6]. This study identified that serum miR-148a expression was upregulated in ICP. Furthermore, *in vivo* research has also confirmed that estradiol upregulated miR-148a expression and miR-148a silencing inhibited TBA secretion induced by estradiol, indicating that miR-148a mediated the effect of estradiol on TBA secretion in LO2 cells. Therefore, this research first demonstrated the regulatory effect of miR-148a on ICP.

PXR, one of the nuclear receptor superfamily members, regulates an entire network of genes which are relate with the detoxification and elimination of xenobiotics, such as the oxidation, conjugation as well as transport [26]. Additionally, PXR plays an important role in bile acid metabolism via regulating several genes expression [26]. Activation of PXR has been shown to protect against cholestatic hepatotoxicity, and one well-known PXR activator, rifampin, is used clinically for the treatment of complications associated with cholestatic diseases [27]. Moreover, promoter DNA methylation of PXR modulates the ICP phenotype [28]. The present study reported that PXR was downregulated by estradiol and the PXR agonist rifampin inhibited TBA secretion induced by estradiol, suggesting a role of PXR in ICP development. On the other hand, miR-148a is reported to bind directly to the 3′-untranslated region of PXR mRNA and inhibit the promotor activation of PXR expression [14]. The present study found that silencing miR-148a expression inhibited the estradiol-induced downregulation of PXR expression. These results demonstrated that as the direct target gene of miR-148a, PXR mediated the effect of miR-148a in ICP development induced by estrogen.

MRP3 is a new ATP-binding cassette protein localised to the canalicular domain of the hepatocyte [16]. MRP3 does not have a major role in bile salt physiology but is involved in the transport of glucuronidated compounds, which could include glucuronidated bile salts in humans [29]. Interestingly, in other studies in human and rat hepatocytes, MRP3/Mrp3 is strongly upregulated under conditions of cholestasis and MRP2 deficiency [30]. The present study showed that MRP3 expression was upregulated in LO2 cells by estradiol. Both miR-148a-siRNA and the PXR agonist rifampin reversed the effect of estradiol on MRP3 expression. These results demonstrated that MRP3 may be involved in ICP development mediated by the miR-148a/PXR signalling pathway indirectly.

However, there were several limitations in this manuscription. Firstly, the luciferase experiments should be performed to identify the direct action of miR-148a on PXR protein expression. Furthermore, in this research, MRP1 and MRP2 should be studied together, while only MRP3 was involved in this research. Therefore, these limitations will be solved in the future research.

## Conclusion

In conclusion, the present study demonstrates that estrogen may induce ICP development via the miR-148a/PXR signaling pathway, and MRP3 may be involved.

## Supporting information

S1 Fig The the uncropped original versions of the Western blots. (A) The presentative image of PXR protein. (B) The presentative image of MRP3 protein. (C) The presentative image of β-actin protein.
(DOCX)

## Abbreviations

ICP: intrahepatic cholestasis of pregnancy
TBA: total bile acid
miRNA: microRNA
PXR: pregnane X receptor
NR: nuclear hormone receptor
LCA: lithocholic acid
ABCA1: ATP-binding cassette subfamily A member 1
MRP3: multidrug resistance protein 3
LV: lentiviral
qRT-PCR: quantitative RT-PCR
siRNA: small interfering RNA
NR1I2: nuclear receptor subfamily 1 group I member 2
