# Supplementary figures and images for: miR-148a-mediated estrogen-induced cholestasis in intrahepatic cholestasis of pregnancy: Role of PXR/MRP3
Source: PLoS One. 2017 Jun 2;12(6):e0178702. doi: 10.1371/journal.pone.0178702 (PMC5457162; doi:10.1371/journal.pone.0178702)

(A)


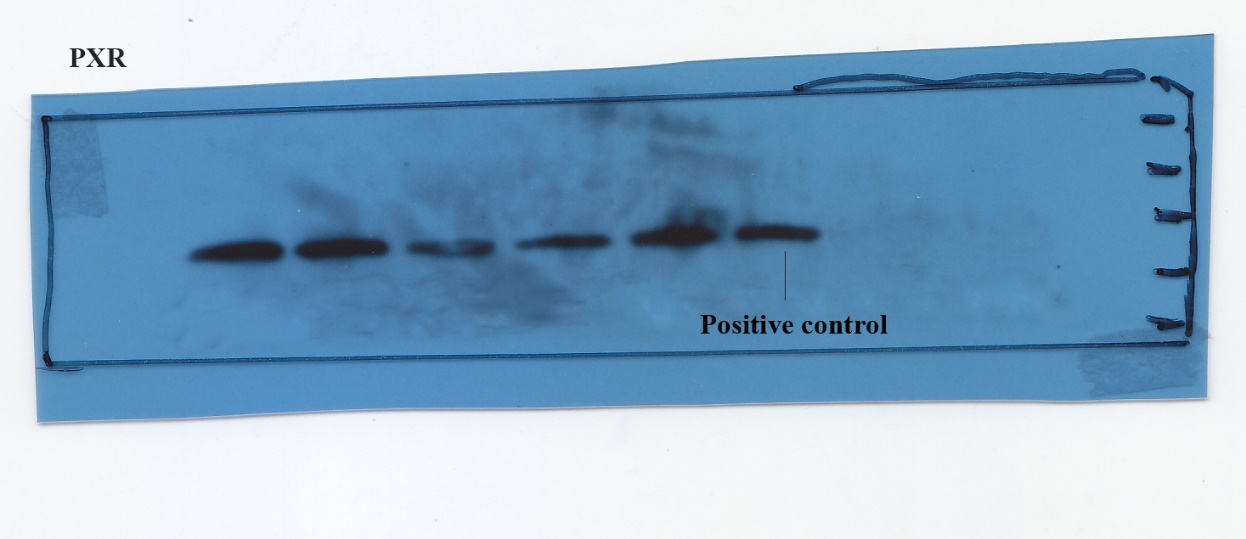


(B)


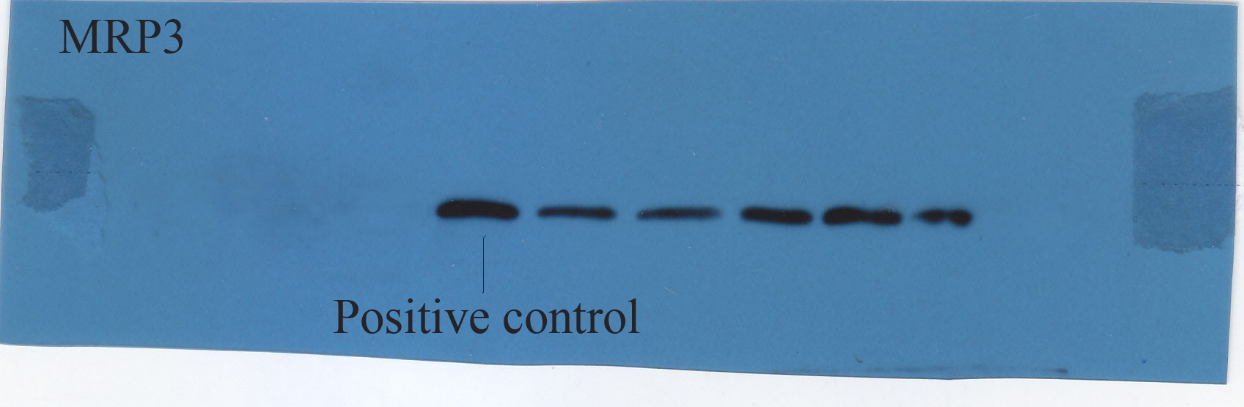


(C)


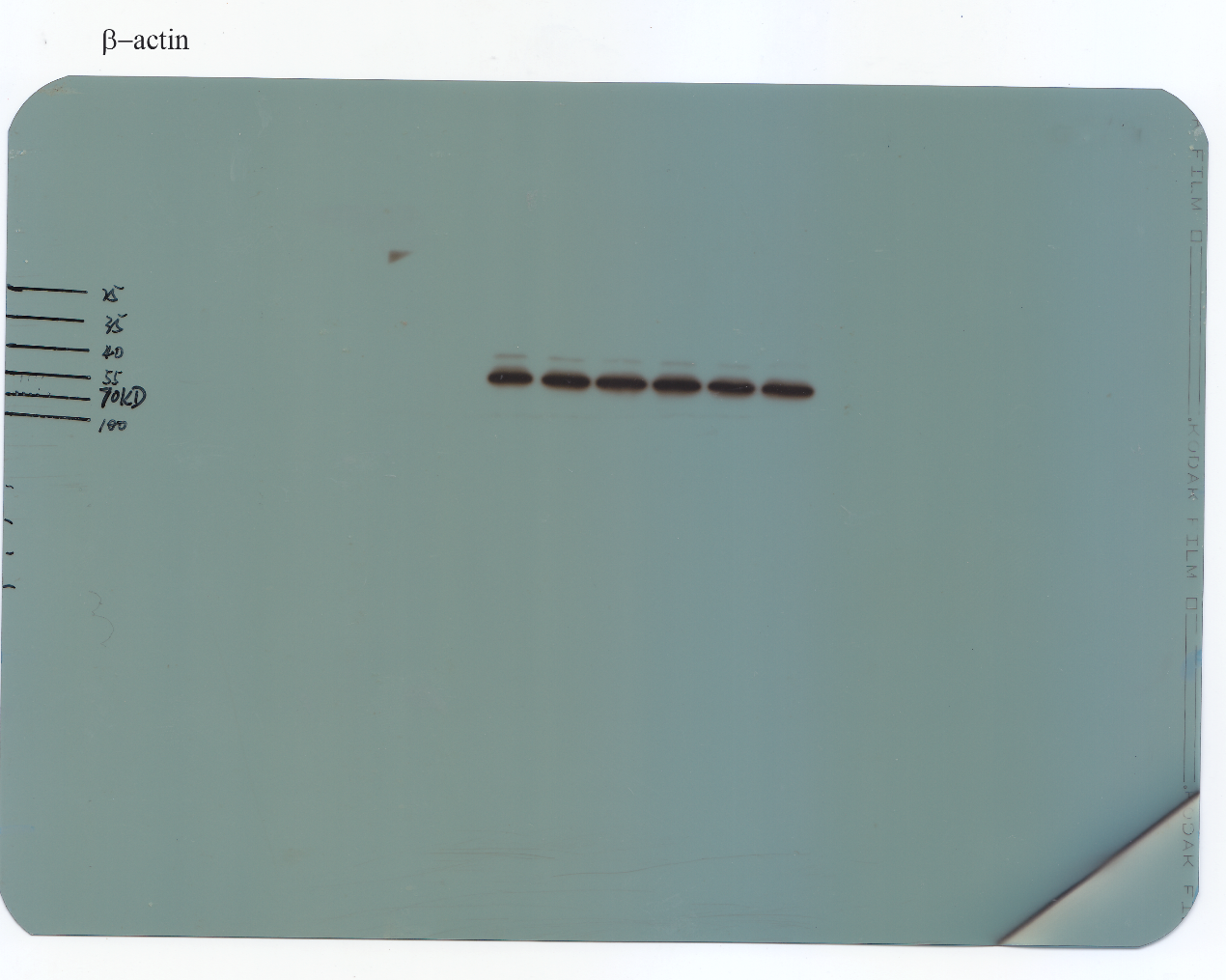


**S1 Fig**

Supplement: S1 Fig — (A) The presentative image of PXR protein. (B) The presentative image of MRP3 protein. (C) The presentative image of β-actin protein. (DOCX) [file pone.0178702.s001.docx]
